# Evaluating shear strength and acoustic emission in rock-like materials with non-persistent joint geometries under freeze-thaw conditions

**DOI:** 10.1038/s41598-025-07943-1

**Published:** 2025-07-01

**Authors:** Sadegh Kefayati, Mosleh Eftekhari, Kamran Goshtasbi, Morteza Ahmadi

**Affiliations:** Department of Mining Engineering, Faculty of Engineering, Tarbiat Modares University, Jalal AleAhmad, Nasr Street, Tehran, 14115-14 Iran

**Keywords:** Rock Bridge, Failure pattern, Acoustic emission, Shear strength, Freeze-Thaw cycles, Engineering, Civil engineering

## Abstract

This study examines the effects of freeze-thaw cycles and the geometric configuration of non-persistent joints on the shear behavior of rock masses. Various artificial rock samples with non-persistent joints underwent direct shear testing to investigate how freeze-thaw cycles (F-T), the rock bridge angle (β), the number of joints (N), and normal stress (σ_n_) influence shear strength and fracture development. Taguchi’s method was employed for experimental design, and the impact of the parameters was evaluated using analysis of variance (ANOVA). Additionally, acoustic emission (AE) detection was utilized to reveal the fracturing characteristics of rock bridges during the tests. The results indicate that normal stress has the most significant effect on shear strength, while the number of joints has the least impact. The angle of the rock bridge is the second most crucial factor influencing shear strength; specifically, low angles lead to tensile failure, while higher angles result in a transition to shear failure. AE data shows that tensile failure occurs at high average frequencies (AF) and low rise angle (RA) values, whereas shear failure exhibits the opposite characteristics. F-T cycles rank third in significance. The results indicate that frost heave primarily affects the specimens in the initial stages of the F-T cycles. Furthermore, the direct shear test results for specimens subjected to F-T cycles are categorized into three stages based on acoustic emission (AE) data: a quiet stage, an AE development stage, and a drop AE stage. Notably, as the number of F-T cycles increases, both the duration of the AE development stage and the AE energy level decrease.

## Introduction

Cold regions encompass significant portions of the Earth, and mining and engineering projects in these areas often face challenges arising from the cycles of freezing and thawing^1–3^. In rock structures, including slopes, tunnels, shafts, and caverns, the deterioration of rock properties due to repeated freezing and thawing is primarily attributed to the thermal expansion and contraction of mineral components, along with the expansive forces generated during the volumetric changes associated with the transition from water to ice within the rocks and their fractures^4^. The increase in pressure encourages the formation of micro-cracks, which can promote the emergence of macro-cracks, significantly impacting the shear behavior of joints within the rock mass. The mechanical and engineering properties of rock masses are greatly influenced by their joints, rendering the shear characteristics of these joints particularly important. In conditions experiencing freeze-thaw (F-T) cycles, joints are particularly susceptible to these effects, leading to a decrease in the shear strength of the rock mass and potentially resulting in failures such as collapses, landslides, avalanches, and rockfalls^5–7^. Therefore, understanding the implications of freeze-thaw cycles on rock masses in cold regions is crucial for ensuring the safety, efficiency, and longevity of mining operations and civil engineering projects, especially considering their impact on shear strength and the associated risk of structural failures.

While numerous studies have been conducted on this topic, most have concentrated on the impact of F-T cycles on the physical^8–13^ and mechanical properties of intact rock^9,14–21^ or the shear resistance of persistent joints^22–26^ Despite the significance of the topic, there are limited investigations into the shear behavior and cracking mechanisms, particularly the microcracking phenomena in rocks with non-persistent joints affected by weathering processes resulting from F-T cycles.

Lei et al.^1^ investigated the shear characteristics of non-persistent joints subjected to F-T cycles using direct shear tests. Their findings indicated minimal effect of freezing and thawing on the shear strength of joints with low persistency. Additionally, they observed a nonlinear reduction in the shear strength of the jointed specimens as the number of F-T cycles increased, with a significant decline in strength occurring after these cycles. Wang et al.^27^ further explored the consequences of F-T fatigue on crack coalescence in granite samples featuring two non-parallel flaws during uniaxial testing. Their research highlighted that the number of F-T cycles and the inclination of flaws significantly influence crack propagation paths, overall sample strength, and deformation behavior. Notably, they observed that under severe F-T damage, the integrity of the rock bridge structure deteriorates, with cracks originating from the tips of the flaws. Qiao et al.^28^ conducted uniaxial compression tests to investigate crack propagation and coalescence in rock samples subjected to varying F-T cycles and rock bridge angles. Their study categorized cracks induced by F-T treatment and compression into main and secondary cracks, revealing that the severity of damage escalates with an increase in F-T cycles, particularly impacting areas around the rock bridges. Daxing et al.^29^ focused on the shear behavior and damage mechanisms in jointed rocks under F-T conditions, utilizing shear tests to analyze microcracking and variations in mechanical properties. Their results indicated that damage intensifies with increased F-T cycles and higher joint persistency, establishing a clear relationship between mesoscopic damage and macroscopic mechanical responses.

The formation of microcracks is a significant early indicator of rock failure, which may occur through macrocracks’ initiation, growth, and coalescence. In laboratory settings, the initiation of microcracks generates elastic waves due to the rapid release of strain energy, a phenomenon referred to as acoustic emission (AE). Analyzing AE parameters and localizing events within AE signals can provide valuable insights into microcracking phenomena by revealing these events’ temporal and spatial progression. Several research efforts have examined the properties of AE, specifically AE counts and energy levels, in certain types of rocks that have undergone different cycles of freeze-thaw processes^27,28,30–38^. Wang et al.^27^ employed AE detection on granite samples subjected to F-T cycles to classify cracks into six distinct types based on the spectral frequency of the AE signals. Their research demonstrated that high-amplitude, low frequency signals serve as reliable predictors of brittle fractures, highlighting the potential of AE in fracture diagnosis. Chen et al.^31^ explored the mechanisms of microcracking in red sandstone subjected to varying cyclic freeze-thaw treatments. Their findings revealed that while the occurrence and distribution of AE events remained relatively stable during the microcracking process under mode I loading, there was a significant reduction in the size and density of these events after exceeding a specific threshold of F-T cycles. This suggests that the material’s response to cyclic loading undergoes notable changes beyond a critical point. Zhang et al.^34^ also investigated the AE signal characteristics of rocks undergoing F-T cycles during shearing processes. Their research indicated that as the number of F-T cycles increased, the differences in AE features across various stages of shearing diminished. This outcome suggests a complex interplay between crack evolution and mechanical loading, underscoring the AE method’s efficacy in monitoring and characterizing material behavior under environmental effects. Liang et al.^39^ conducted quasi-static compression experiments on fissured sandstone subjected to F-T cycles, employing acoustic emission (AE) monitoring to evaluate failure characteristics. The study demonstrated that F-T cycles and the angles of fissures significantly influence failure patterns by increasing internal structural defects and altering the distribution of stress conditions. Additionally, they noted that the Rise Angle (RA) and Average Frequency (AF) values reflect the progression of various crack types.

Most previous studies have focused on uniaxial specimens, whereas direct shear tests offer more accurate results by simulating the real conditions of non-persistent joint failure.

**Fig. 1:**
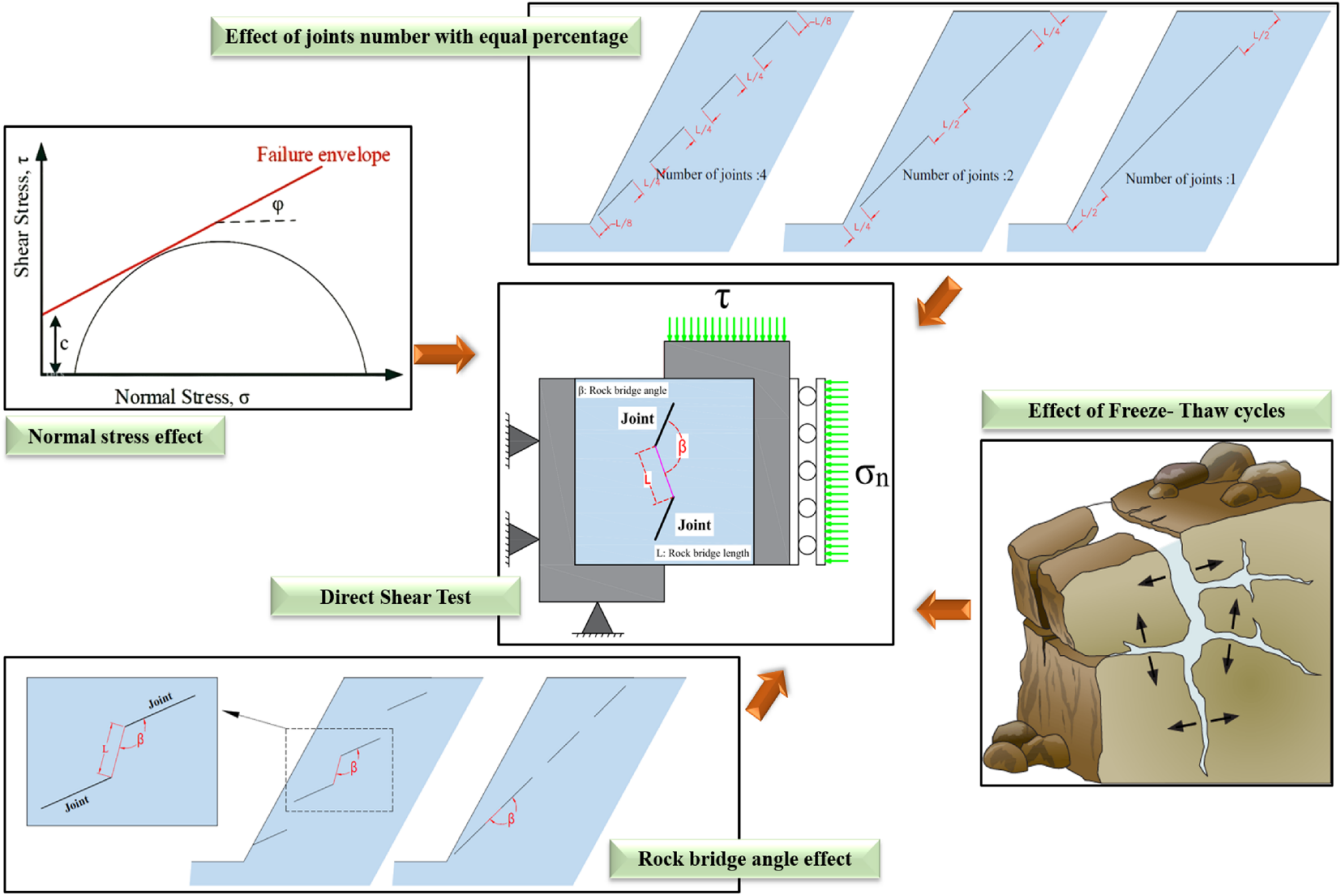
Investigating the effect of four parameters on the shear strength of rock bridge in direct shear test.

Furthermore, they indicate that the varying lengths of joints influence the shear strength of rock bridges, and that the angles of these rock bridges affect the type of failure exhibited. The interaction between these two parameters and their effects on shear behavior and crack growth becomes more complex when considering the impacts of F-T cycles. Therefore, it is crucial to thoroughly understand the rock bridge shear behavior in different geometries with mechanisms and progression of freeze-thaw damage related to the water/ice phase transition in rocks and their discontinuities. In Fig. 1, the total length of the rock bridge in three cases is (L), although each case exhibits a distinct pattern of the rock bridge. This variation can influence the slope’s shear behavior. Moreover, this figure illustrates how the dip of the joints leads to differing angles of the rock bridges relative to one another. These angles are critical, as they impact the mechanisms of crack development and slope failure between the joints. The primary objective of this study is to investigate how the number of joints with an equal percentage of rock bridge (K) at various angles (β) under differing normal stresses affects the direct shear strength of rock, particularly under the influence of F-T cycles (see Fig. 1). Furthermore, this study employs acoustic emission methods to analyze the formation and coalescence of microcracks, thereby providing valuable insights into the behavior of rock under shear stress.

Based on the above explanation, this research addresses a significant gap in the existing literature, as the interactions among these factors and their effects on the shear strength of rock have received insufficient attention. The results of this investigation enhance our understanding of shear strength, failure mode prediction, and the monitoring and assessment of the structural integrity of rock masses with non-persistent joints influenced by different F-T cycles. This knowledge facilitates more precise designs of rock engineering structures, especially in cold regions where these phenomena frequently occur.

## Experimental setups

### Specimen preparation

Given the challenges and significant expenses associated with prefabricating joints in rock, researchers often employ artificial rock specimens to study the influence of joints on the mechanical strength properties of rock^40,41^. This study utilized cement mortar, a rock-like material, to create the specimens. The mixing ratio was 38% cement, 38% plaster, and 24% water. The physical and mechanical parameters of the rock-like specimen are shown in Table 1.

**Fig. 2:**
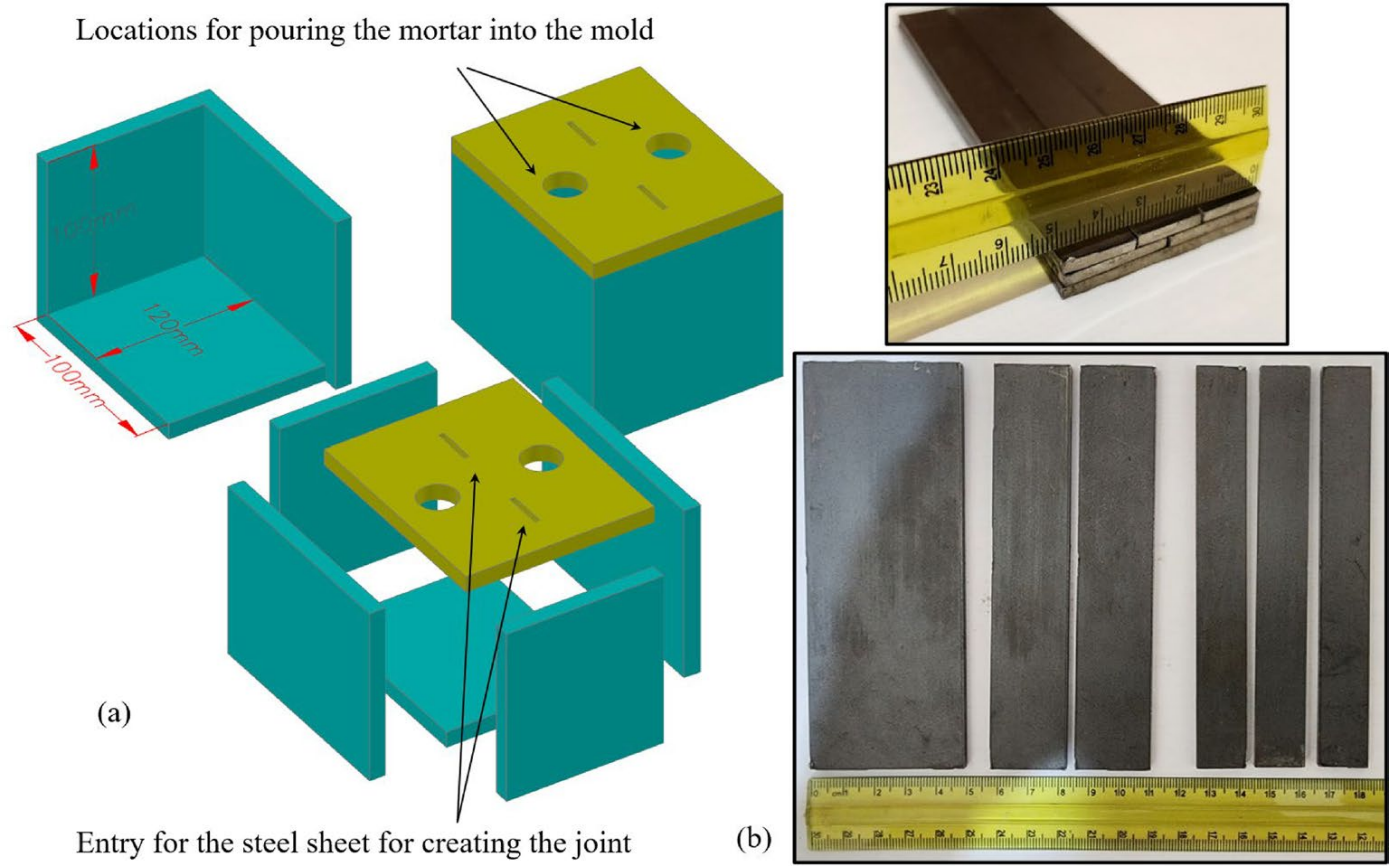
(**a**) Mold geometry for preparing artificial rock specimens, (**b**) Steel sheets for making joints in artificial rock specimens.

**Fig. 3:**
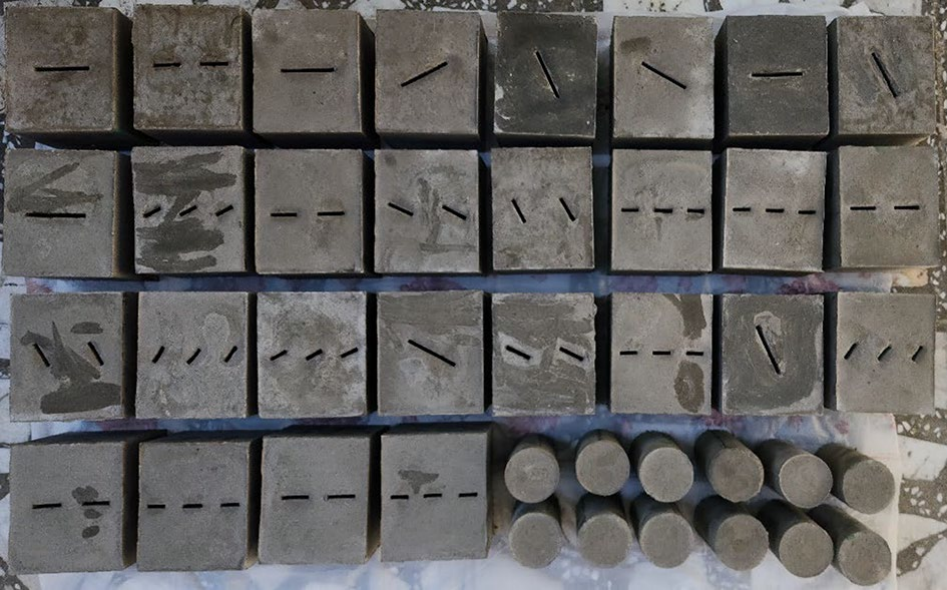
Some rock-like specimens for direct shear, uniaxial, and tensile strength tests.

The mold dimensions are 120 mm in width, 100 mm in length, and 100 mm in height (see Fig. 2a). Steel sheets, as shown in Fig. 2b, create prefabricated cracks inside the specimens. The steel sheets measure 100 mm in length, 3 mm in thickness, and widths of 16.67 mm, 25 mm, or 50 mm. Following one hour of solidification, the steel sheets are removed to create the prefabricated cracks, and the specimens are then extracted from the mold after a total solidification period of 20 h. Finally, the specimens were placed in a curing chamber for 28 days to complete their preparation. In the testing procedure, several artificial specimens with one, two, and three non-persistent joints, each maintaining an equal percentage (K) at bridge angles of 90, 135, and 180 degrees, were subjected to direct shear testing. Additionally, cylindrical specimens were prepared for uniaxial and tensile tests to evaluate the effects of F-T cycles (see Fig. 3). To ensure a sufficient number of experiments and a robust statistical understanding, a statistical framework based on the Taguchi method^42^ was utilized.

**Table 1:** Rock-like specimen’s properties.

Density	Porosity	Uniaxial strength	Tensile strength	Elastic modulus	Poisson’s ratio	*P*-wave velocity
(Kg/m^3^)	%	(MPa)	(MPa)	(GPa)		(m/s)
1960	10.1	17.2	2.5	3.38	0.2	2194

### Taguchi method

Taguchi and Konishi^42^ proposed a three-phase approach to optimizing the necessary number of experimental trials. The initial phase involves identifying which input parameters significantly impact the output or response variables, such as the number of joints (N), rock bridge angle (β), F-T cycles, and normal stress (σ_n_) in this research. In the subsequent phase, nominal levels for these parameters are established using an orthogonal array (OA), where each parameter corresponds to a specific column, and each row represents a particular set of values related to these parameters. It is common for some columns to remain vacant to account for potential errors or interactions among the parameters. Standard examples of orthogonal arrays include L4, L9, L16, and L27, where the number associated with each array indicates the total number of experiments to be performed. The next phase focuses on designing tolerances to reduce variability in the statistically significant parameters. Ultimately, the contribution of each input variable (for instance, N, β, F-T cycles, and σ_n_ in this investigation) to the output variable (such as τ of non-persistent jointed rocks) is assessed through the application of analysis of variance (ANOVA). For those interested in the mathematical foundations of ANOVA, references such as^43^ provide comprehensive information. In this research, the ANOVA analysis was performed using Minitab software.

In the Taguchi method, repetition plays a crucial role in ensuring the reliability and validity of experimental results. Researchers can account for variability and minimize random errors by conducting multiple trials for each experimental setup. This approach allows for a more accurate estimation of the effects of different factors on the response variables. Furthermore, repetition enables the identification of consistent patterns within the data, enhancing the robustness of conclusions drawn from the analysis. Overall, incorporating repetition into the experimental design fosters greater confidence in the findings and supports the generalizability of the results.

**Fig. 4:**
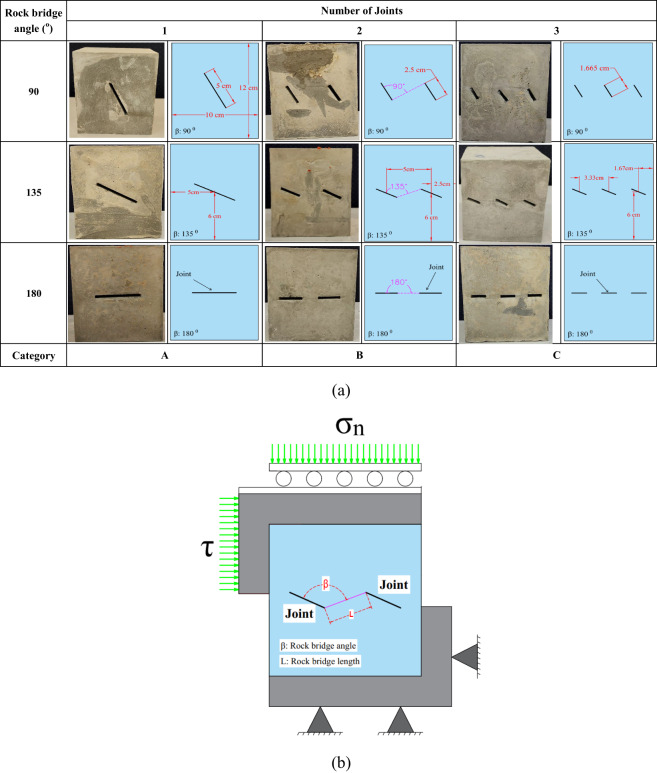
(**a**) Arrangements of non-persistent joints utilized in the direct shear testing, (**b**) Schematic of the direct shear test.

In this study, to investigate the influence of four parameters on shear strength- namely, the number of joints (maintained at a constant percentage (K)), the rock bridge angle (β), F-T cycles, and normal stress (σ_n_) - each assessed at three distinct levels, an L27 orthogonal array was generated by the Taguchi Design of Experiments (DoE) methodology. All parameters and their corresponding levels are summarized in Table 2. The influence of joints on the slope stability of roads and open-pit mines is significant at the bench scale, mainly due to their potential to create unstable blocks. This study applied normal stress values of 0.1, 0.3, and 0.5 MPa to reflect conditions at the bench scale. Three values- 90 degrees, 135 degrees, and 180 degrees- were used for bridge angles, respectively, representing overlapping, intermediate, and non-overlapping states. The F-T cycles considered were 0, 10, and 20, allowing for the evaluation of samples in a non-freeze-thaw condition as well as under 10 and 2 × 10 F-T cycles. The number of joints varied between 1, 2, and 3, facilitating the assessment of the effect of a single large rock bridge compared to multiple smaller rock bridges positioned in the same area but of equal length, while maintaining a consistent total joint length of 5 cm across all cases. Figure 4 illustrates all patterns of non-persistent joints relevant to this investigation.

**Table 2:** Specified parameters and levels for the experimental design using the Taguchi method.

Parameters	Symbol	Level		
		1	2	3
Normal stress (MPa)	σ_n_	0.1	0.3	0.5
Cycle (freezing-thawing)	F-T	0	10	20
Bridge angle (degree)	β	90	135	180
Joint number	N	1	2	3

### Experimental equipment and procedure

**Fig. 5:**
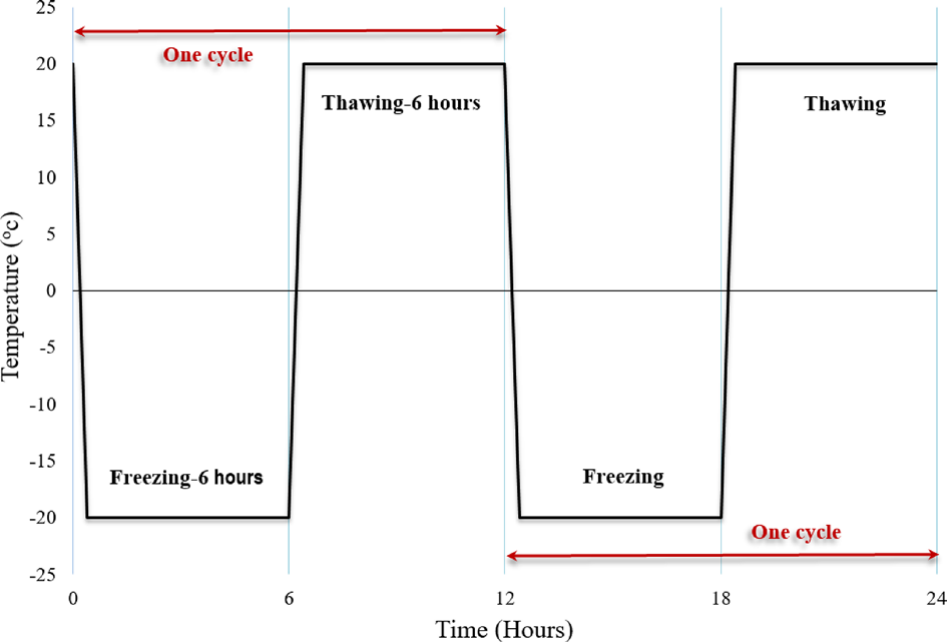
Schematic representation of the F-T cycle.

Before the direct shear test, several preparatory steps were conducted on the specimens. First, the specimens were dried, followed by a P-wave velocity assessment and weight measurement. Subsequently, the specimens were saturated using the boiling method. The saturated specimens were then encased in plastic film, and the prefabricated cracks were filled with pure water. The mass of the specimens in the saturated condition was recorded. The saturated joint specimens were placed in a freeze-thaw chamber, frozen at −20 °C for 6 h, followed by thawing at 20 °C for an equivalent period (see Fig. 5). Each freeze-thaw cycle lasted 12 h. The specimens were categorized into three groups based on the number of cycles: 0, 10, and 20. Upon completion of the freeze-thaw cycles, the sample is weighed to ensure that no water is lost during the process. In other words, the weight should match the saturated sample before the freeze-thaw cycles. This confirms that all water in the sample has participated in the freezing and thawing process. Finally, the samples were dried in an oven at 80 °C for 420 min, after which their drying mass and P-wave velocity were measured again.

Furthermore, during the direct shear test, AE technology was employed to investigate the effects of the parameters (N), (β), and F-T cycles on the behavior of microcracks. The shear rate in the direct shear tests was 0.006 mm/s.

**Fig. 6:**
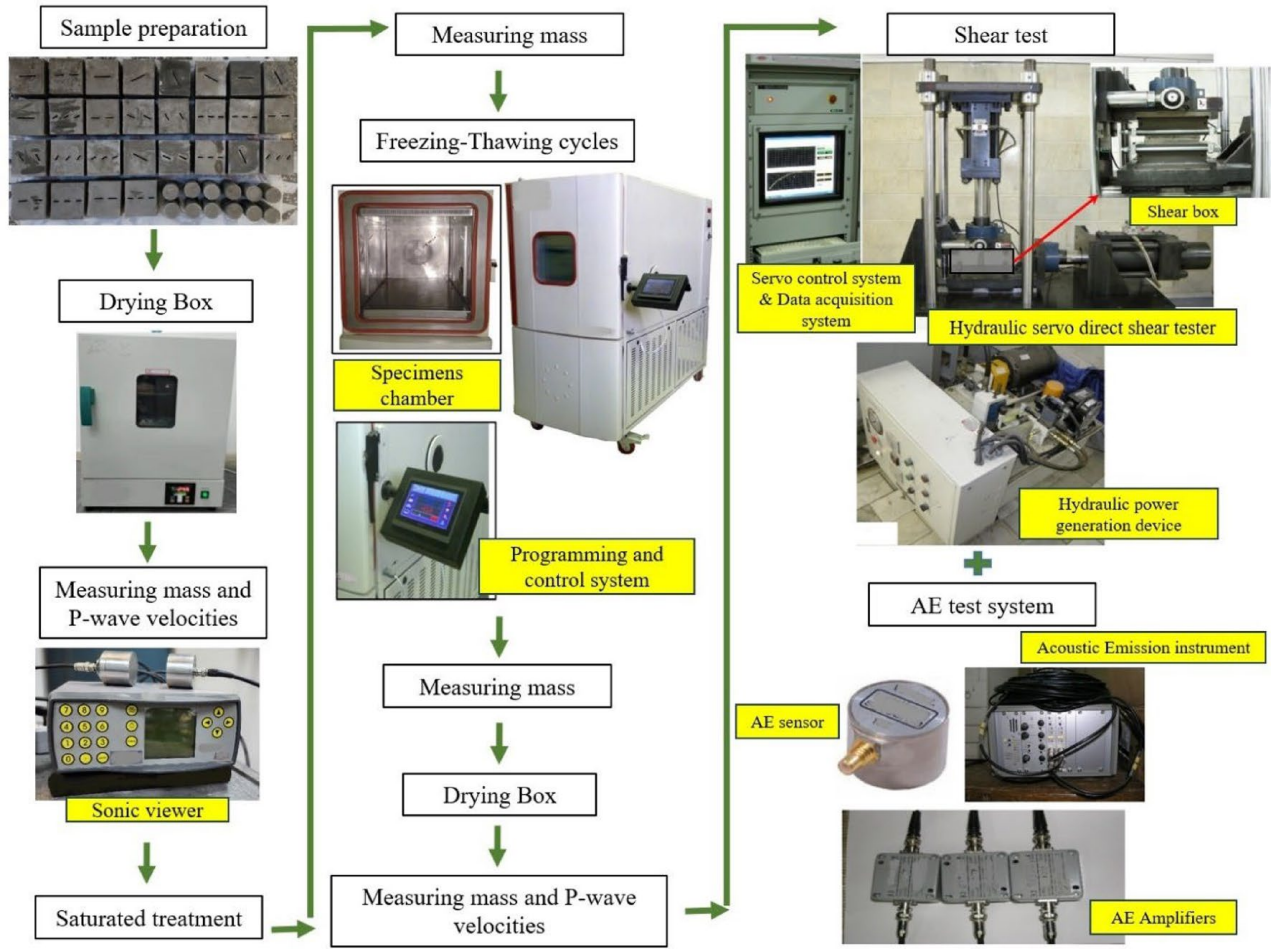
Schematic of experimental equipment and procedures.

The main equipment used in this investigation is shown in Fig. 6.

## Experimental results

### Analysis of physical characteristics

**Fig. 7:**
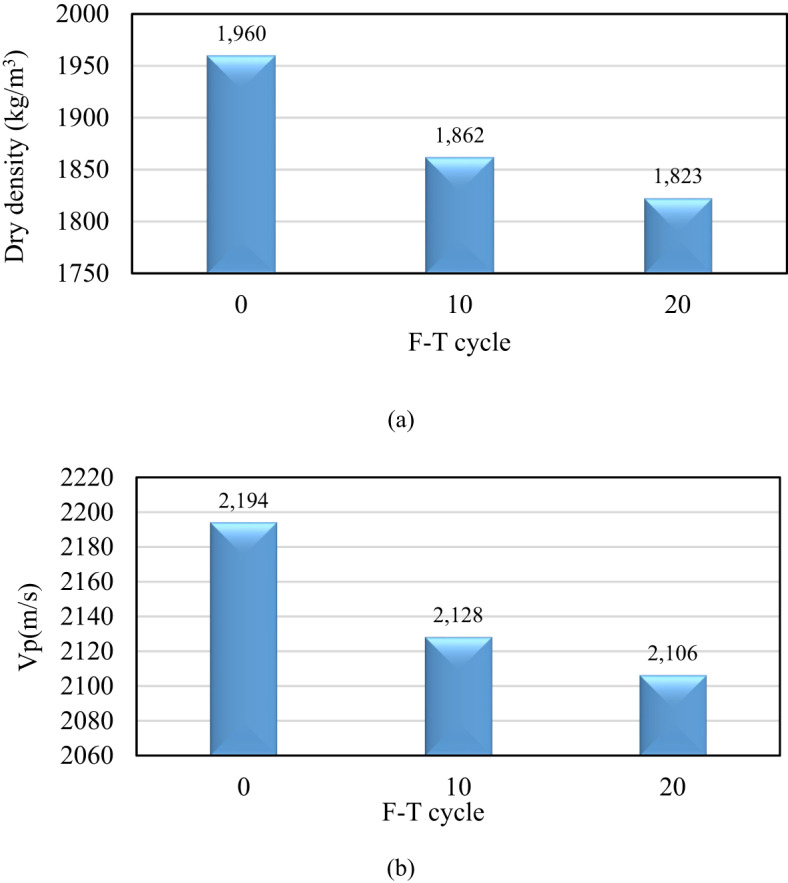
(**a**) Dry density of samples following various F-T cycles, (**b**) P-wave velocity measurements of samples before and after exposure to F-T cycles.

Figure 7a illustrates the dry density of the specimens subjected to various F-T cycles. The data indicates a reduction in density of approximately 5% following 10 F-T cycles, with a further decrease of around 7% observed after 20 cycles. In a similar trend, the P-wave velocity mirrors the changes seen in density. As displayed in Fig. 7b, a 3% decrease in P-wave velocity is noted after 10 F-T cycles, escalating to a 4% reduction after completing 20 cycles.

The volume expansion resulting from water’s phase transition leads to an increase in the size and quantity of pores within the specimens due to the pressure exerted by frost heave. As the number of F-T cycles increases, the integrity of the internal structure experiences further degradation, which can result in the fragmentation of rocks or a decrease in their quality.

**Fig. 8:**
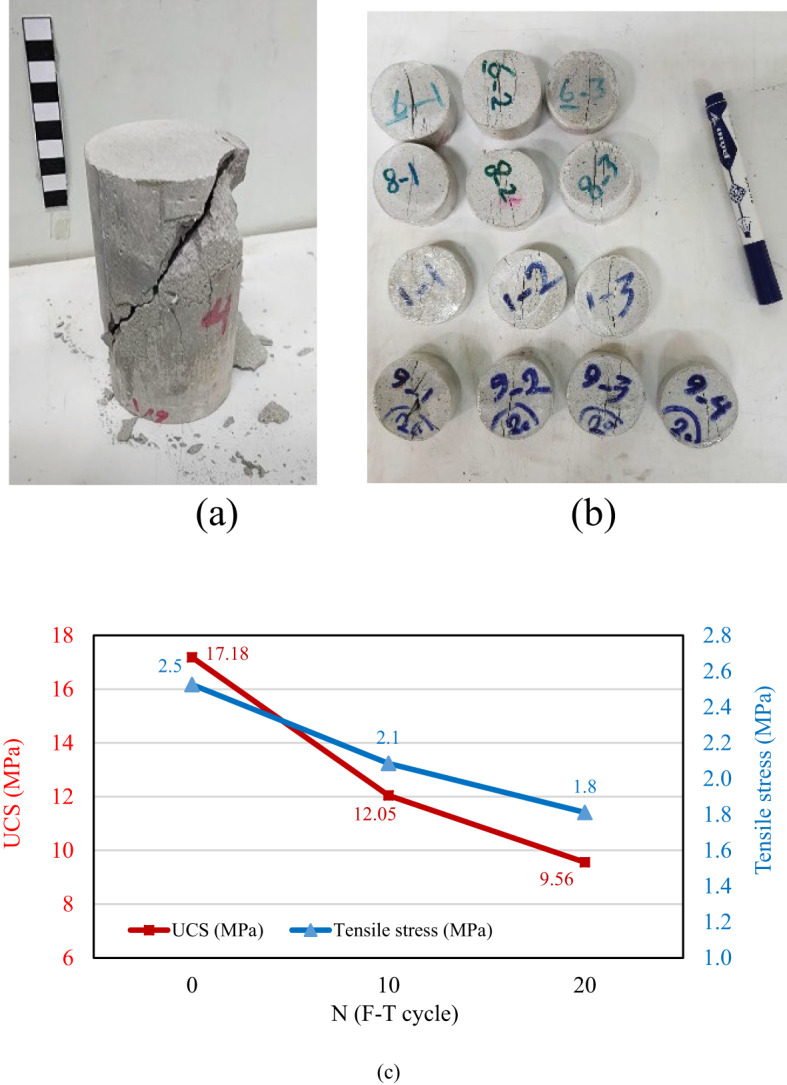
(**a**) UCS Test, (**b**) Brazilian Test, (**c**) Variation in compressive and tensile strength with the increase in F-T cycles.

A series of uniaxial and Brazilian tests were performed to assess the mechanical properties of rock-like specimens according to ISRM^44^. The findings indicate that as the number of F-T cycles increases, there is a reduction in both compressive and tensile strength, with the most decline occurring during the initial ten cycles (see Fig. 8). The specimens examined contain many pores and flaws. Repeated freeze-thaw (F-T) cycles generate frost heave pressure, leading to macro-scale damage to these defects, the formation of secondary cracks, and facilitating water movement. Water within the cracks gradually migrates to the frozen areas, where it freezes. Furthermore, microcrack water can only overcome gravity and migrate into the frozen regions when the crack length is less than 1 μm during the F-T process^45^. Consequently, the repeated action of frost-induced stress and crack propagation occurs during the F-T cycle for microcracks exceeding 1 μm in length.

In contrast, for microcracks shorter than 1 μm, unfrozen water within the rock tends to migrate toward larger macrocracks. This migration leads to the expansion of macrocracks under frost stress, while microcracks may close due to water movement. Since the water content in the tested specimens is constant throughout the F-T cycles, the extension of macrocracks during the initial F-T cycles creates additional space, reducing freezing pressure. Therefore, the damage to the specimens caused by frost heave is primarily concentrated in the early stages of the F-T cycles. Previous research has obtained similar results^9,28^.

### Influence of various factors on shear strength and failure behavior

Based on the Taguchi Design of Experiments (DoE) methodology, twenty-seven direct shear tests were conducted on specimens characterized by non-persistent joints, with three repetitions, as detailed in Table 3. Analysis of variance (ANOVA) was used to study the effect of each parameter on shear strength. According to the data presented in Table 4, parameters exhibiting a higher percentage contribution demonstrate a more significant impact on shear strength. Specifically, normal stress (σ_n_), which corresponds to the highest percentage contribution, has the most significant influence on shear strength, while the number of joints (N) ranks last in terms of effect. Additionally, the angle of the rock bridge (β**)** and the F-T cycles are the second and third positions regarding their influence, respectively.

Figure 8 presents the signal-to-noise (SN) ratio for each analyzed parameter at various levels. The mean SN ratio is an important statistical tool for assessing how different parameters affect experimental outcomes. This method is particularly beneficial in optimization studies, including those based on the Taguchi approach. By calculating the mean of the SN ratios for each parameter, researchers can effectively evaluate the influence of each factor on the results. A main effect occurs when varying levels of a factor lead to different influences on the outcome being studied. As shown in Fig. 8, a notable main effect is indicated when the line plotted is not horizontal, signifying that it deviates from parallel to the x-axis. The degree of this vertical deviation among the data points illustrates the magnitude of the main effect; a larger difference indicates a more significant impact.

**Table 3:** Experiments designed by Taguchi method and results of direct shear tests.

Test No.	Parameters				Result
	σ_*n*_ (MPa)	F-T (Cycle)	β (degree)	*N* (number of joints)	τ (MPa)
1	0.1	0	90	1	0.46
2	0.1	0	90	1	0.53
3	0.1	0	90	1	0.49
4	0.1	10	135	2	0.55
5	0.1	10	135	2	0.50
6	0.1	10	135	2	0.59
7	0.1	20	180	3	0.49
8	0.1	20	180	3	0.58
9	0.1	20	180	3	0.53
10	0.3	0	135	3	0.63
11	0.3	0	135	3	0.60
12	0.3	0	135	3	0.79
13	0.3	10	180	1	0.64
14	0.3	10	180	1	0.69
15	0.3	10	180	1	0.79
16	0.3	20	90	2	0.54
17	0.3	20	90	2	0.56
18	0.3	20	90	2	0.51
19	0.5	0	180	2	1.04
20	0.5	0	180	2	0.95
21	0.5	0	180	2	1.20
22	0.5	10	90	3	0.89
23	0.5	10	90	3	0.79
24	0.5	10	90	3	0.85
25	0.5	20	135	1	0.89
26	0.5	20	135	1	0.99
27	0.5	20	135	1	1.10

**Table 4:** Analysis of variance (ANOVA) results.

Source	DF	Sum of squares	Mean square	Percentage contribution
δ_n_ (MPa)	2	0.94829	0.474144	80.30%
F-T (Cycle)	2	0.01556	0.007778	1.32%
β(degree)	2	0.10287	0.051433	8.71%
N	2	0.01069	0.005344	0.91%
Error	18	0.1036	0.005756	8.77%
Total	26	1.181		100%

**Fig. 9:**
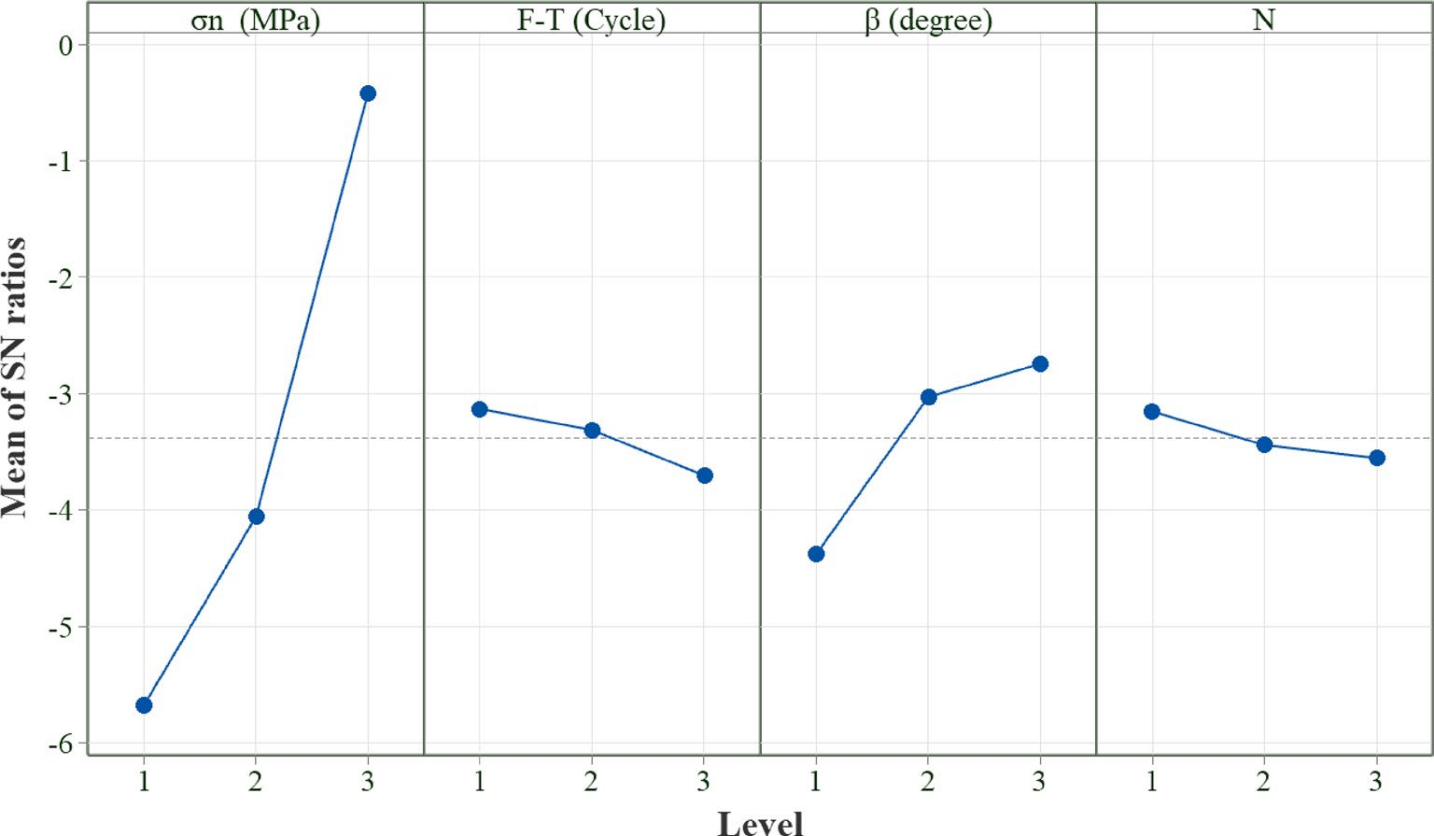
The effect of parameters on the shear strength based on SN ratios.

#### Influence of normal stress

Normal stress plays an important role in influencing the shear strength of rock bridges. Overall, increasing normal stress typically increases shear strength, effectively enhancing the interlocking of mineral grains and promoting frictional resistance. In this investigation, the finding indicates a significant influence of normal stress on shear strength. Figure 9 illustrates that as the normal stress escalates from 0.1 MPa to 0.3 MPa and subsequently to 0.5 MPa, there is a corresponding increase in shear strength. This can be attributed to the gradual enhancement of frictional forces along the surface of the joints, which helps to diminish stress concentration around the tips of the rock bridges, thereby enhancing the overall shear strength of the test specimen. This outcome agrees with earlier researchers’ findings^46^.

#### Influence of rock bridge angle

**Fig. 10:**
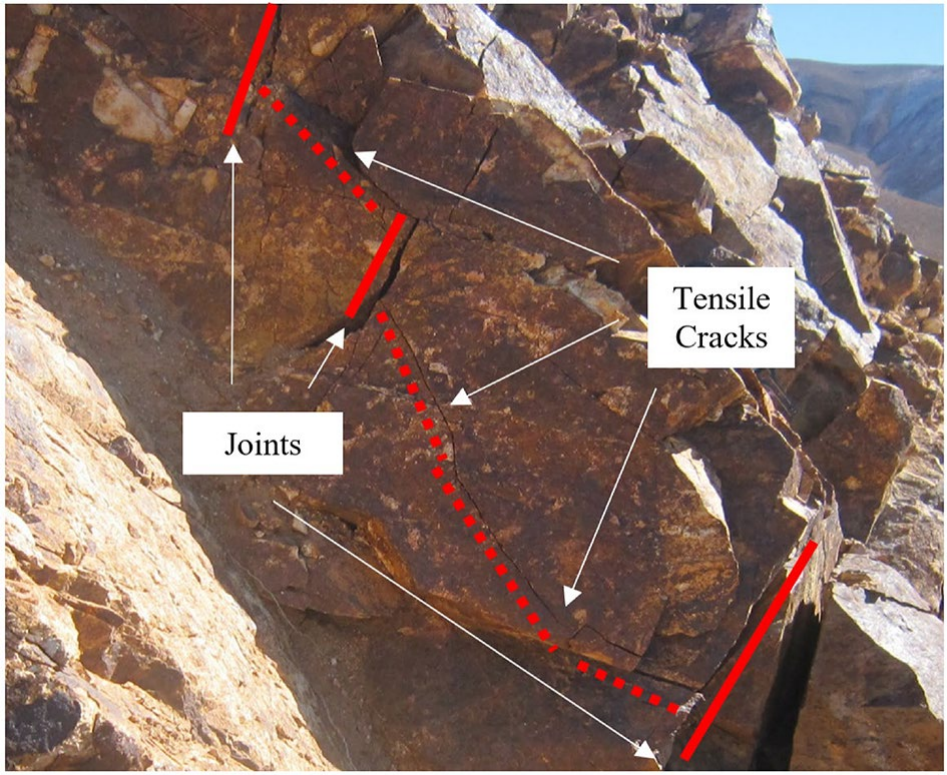
Failure at the rock bridge.

When external forces are applied to the rock mass, the existence of various discontinuities changes the distribution of stress, resulting in diverse failure modes. Figure 10, illustrates failures in rock bridges originating from preexisting overlapping flaws in the rock slope of the copper mine.

As illustrated in Fig. 9, the rock bridge angle has the greatest effect on shear strength after normal stress; as the rock bridge angle increases, the shear strength also tends to increase.

**Fig. 11:**
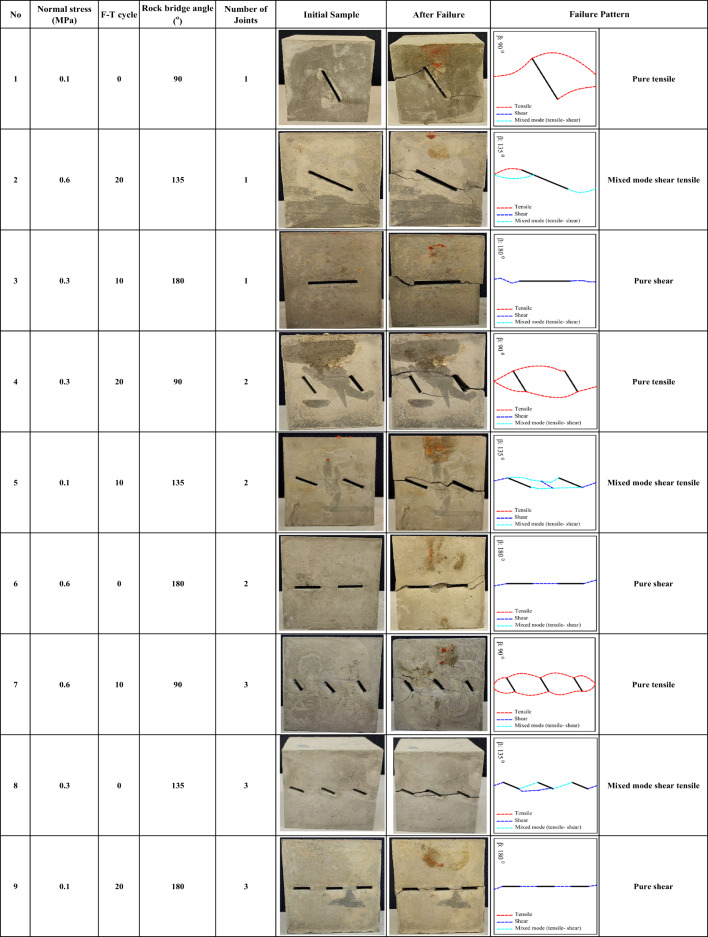
Failure modes of specimens under direct shear test.

Figure 11 illustrates the various failure modes observed in the rock bridge, including tensile failure, shear failure, and mixed failure (shear + tensile), all resulting from variations in the rock bridge angle.

When the rock bridge angle is 90°, wing cracks originate from either the boundary of the specimen or the tips of pre-existing joints. As the shear stress increases, these cracks propagate toward the tips of the opposite joints or propagate at the opposite boundary. These conditions are observed in all three specimens with one, two, and three pre-existing joints, indicating that tensile failure is the dominant mechanism in these specimens. In other words, in overlapping non-persistent joints, the interaction between adjacent joints leads to complex stress distributions. When shear forces are applied, these overlapping regions experience enhanced localized stresses due to the mechanical coupling of the joint surfaces. As shear stress increases, the tensile strength of the rock may be exceeded, leading to the initiation of tension cracks. These cracks typically propagate at an angle close to perpendicular to the direction of the applied shear force. This type of failure in the rock bridge at a large scale within the rock slope of the copper mine is illustrated in Fig. 10.

**Fig. 12:**
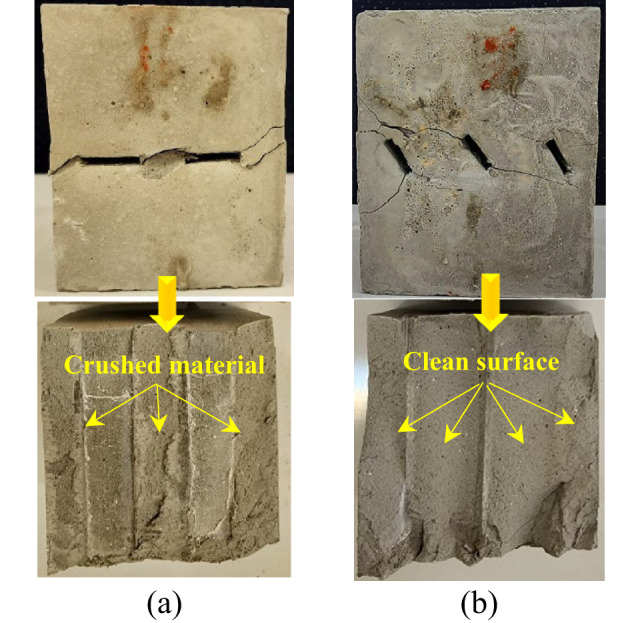
Shear and tensile failure in the rock bridge: (**a**) crushed material on the crack surface in the rock bridge region indicates a shear failure mode, and (**b**) a crack surface devoid of any crushed material signifies a tensile failure mode.

A combination of shear and tensile cracks is observed in the specimens with a 135° rock bridge angle. It is important to note that, in these specimens, shear cracks have a more significant effect on failure than tensile cracks. The third failure category was identified in specimens exhibiting a rock bridge angle of 180 degrees. This failure mode is characterized by shear cracking. In these specimens, shear cracks extend and converge at the bridge region between the tips of the joints. Consequently, the primary factor contributing to failure in these samples is the propagation of shear cracks. A more complete explanation is that in non-overlapping, non-persistent joints, the absence of direct interaction between joints allows for a more uniform stress distribution throughout the rock mass. Under shear loading, the primary mode of crack propagation in non-overlapping joints is shear cracking along the joint surfaces. The applied shear force causes the two rock masses to slide against each other, leading to the formation of distinct shear cracks. These cracks typically align with the joint orientation and are characterized by a sliding mechanism that promotes failure along the joint. In addition to the above explanation, this study utilized the framework established by Wong and Einstein^47^ to investigate the cracking process of the specimens. Furthermore, this study employed another diagnostic method to differentiate shear cracks from tensile cracks by observing the surface characteristics of the cracks at the end of the testing process. Specifically, crushed material is present on the surfaces of shear cracks, whereas such features are absent in tensile cracks Fig. 12.

To enhance the examination of shear and tensile fractures induced by the geometry of the rock bridge during direct shear testing, this research also employs the acoustic emission (AE) technique.

**Fig. 13:**
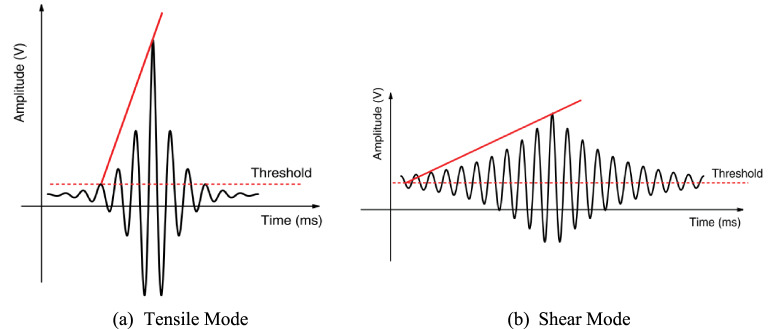
Acoustic Emission Waveform Modes Associated with Tensile and Shear Cracks^48^.

**Fig. 14:**
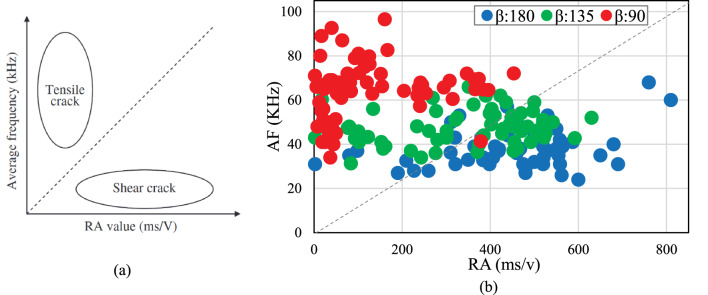
Correlation between AF and RA value, (**a**) crack classification method^51^ (**b**) Crack types in rock specimens at different rock bridge angles.

Each detected AE signal can be linked to a particular type of crack based on AE parameters. The inherent elongation associated with tensile crack propagation induces lateral crack motion, producing short-duration, high-frequency AE waveforms (Fig. 13a). In contrast, shear crack development generates extended waveforms displaying lower frequency components and prolonged rise times (Fig. 13b). This delay may be attributed to the fact that a greater portion of the energy is transmitted as shear waves, which propagate at a slower rate. As a result, the maximum peak of the waveform occurs significantly later than that of the longitudinal waves that arrive first^48,49^. To differentiate these crack types, a parametric analysis is conducted to identify the crack type associated with each AE signal, enabling the differentiation between two primary crack modes: tensile and shear cracks, as illustrated in Fig. 14a. This classification, proposed by the Japan Concrete Institute (JCI) and standardized in JCMS-IIIB5706^50^ relies on the calculation of two additional parameters: Rise Angle (RA) and Average Frequency (AF). These parameters are defined in Eqs. (1) and (2)^51^.

1 $$RA = rise time/ maximum\, amplitude$$

2 $$AF = count/ duration\, time$$

AE analysis, using AF/RA diagrams and energy histograms, effectively examines microcrack behavior and crack types, aligning with established practices in rock mechanics studies^27,28,31^. In this investigation, three direct shear tests were conducted using the acoustic emission (AE) method to assess the influence of the rock bridge angle on the development of shear and tensile cracks, as shown in Table 5. During these tests, data obtained through the AE method were processed to compute the AF and RA values, which were subsequently compared with Fig. 14a. Figure 14b indicates that a specimen with a rock bridge angle of 90^o^ exhibits high AF and low RA values, suggesting the occurrence of tensile cracking. Conversely, at a rock bridge angle of 180^o^, the AF and RA values are reversed compared to the specimen with a 90^o^ rock bridge angle, resulting in shear failure. Furthermore, at a rock bridge angle of 135^o^, the failure process reflects a combination of shear and tensile cracking as observed in the direct shear test.

Another distinction observed in failure types relates to shear strength values. As illustrated in.

**Fig. 15:**
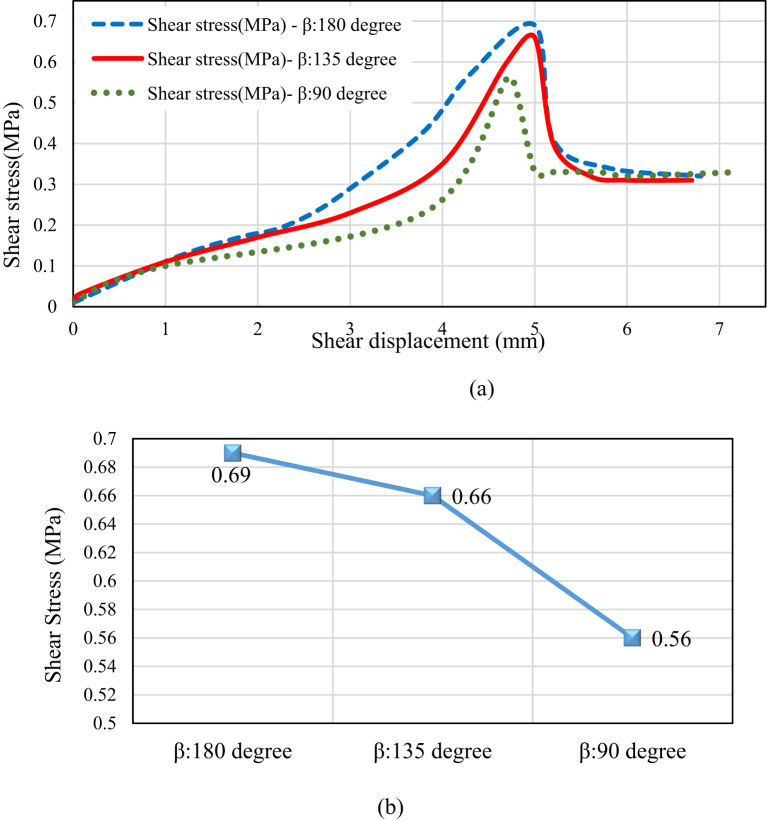
(**a**) Shear stress vs. shear displacement curves, (**b**) Peak shear strength for different rock bridge angles.

Figure 15, the shear strength of the sample with a rock bridge angle of 180° is greater than that of the sample with a 90° angle. Specifically, for the 180° bridge angle, the peak shear resistance value is 0.69 MPa, approximately 23% higher than the peak shear strength value for the 90° angle. Additionally, at a rock bridge angle of 135°, the shear strength reflects a value that suggests a composite failure mode between the two previously mentioned angles. This difference is linked to shear and tensile failure modes, underscoring the importance of the rock bridge angle in creating failure modes.

**Table 5:** Direct shear testing with variable rock Bridge angle.

Test no.	σ_*n*_ (MPa)	F-T (Cycle)	β (degree)	*N* (number of joints)
28	0.3	10	180	1
29	0.3	10	135	1
30	0.3	10	90	1

#### Influence of F-T cycle

Figure 9 illustrates that the F-T cycle is the third factor influencing shear strength in this investigation. As the number of F-T cycles increases, the shear strength of the samples decreases. Samples not subjected to F-T cycles exhibit a more integrated structure with fewer internal micro cracks. In contrast, specimens exposed to F-T cycles develop significant cracks due to the pressures from frost heaving.

In this investigation, the influence of F-T cycles on shear strength and the progression of microcracks during the direct shear test was explored further by conducting additional experiments, as specified in Table 6, alongside the analysis of acoustic emission data.

**Fig. 16:**
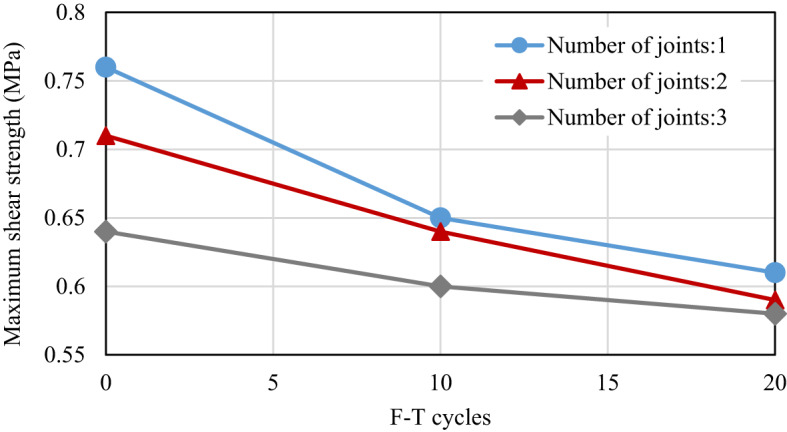
Influence of F-T Cycles on maximum shear strength across various joints.

Figure 16 demonstrates that as the number of F-T cycles increases, shear strength decreases across all tested samples, regardless of their joint configurations. Notably, the most significant decline in shear strength is observed during the first ten cycles. This trend indicates a reduction in peak frost-heaving pressure with increasing F-T events. During the initial F-T cycle, the frost-heaving pressure is substantial, as the jointed specimens have yet to experience any damage from the effects of frost heaving. However, with repeated cycles, this pressure leads to considerable macro-damage to the joints and promotes water migration. Thus, the destruction of joints caused by frost heaving occurs mainly in the early stages of the F-T cycles.

**Table 6:** Direct shear testing with variable F-T cycles and number of joints.

Test No.	σ_*n*_ (MPa)	F-T (Cycle)	β (degree)	*N* (number of joints)
31	0.3	0	180	1
32	0.3	10	180	1
33	0.3	20	180	1
34	0.3	0	180	2
35	0.3	10	180	2
36	0.3	20	180	2
37	0.3	0	180	3
38	0.3	10	180	3
39	0.3	20	180	3

**Fig. 17:**
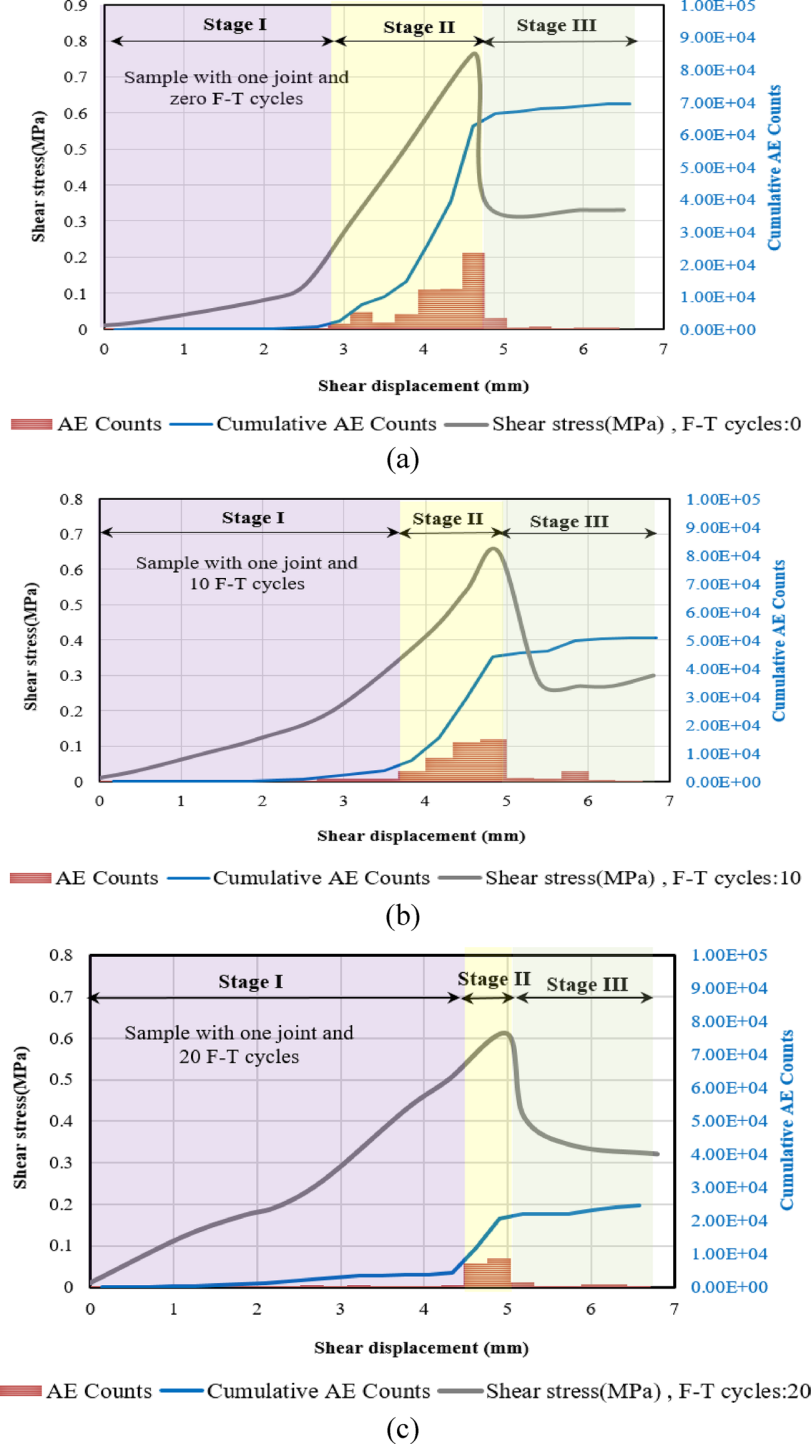
Correlation of shear stress, AE Counts, cumulative AE counts, and shear displacement for samples with one joint subjected to various F-T cycles, (**a**) Sample not subjected to F-T cycles, (**b**) Sample subjected to 10 F-T cycles, (**c**) Sample subjected to 20 F-T cycles.

**Fig. 18:**
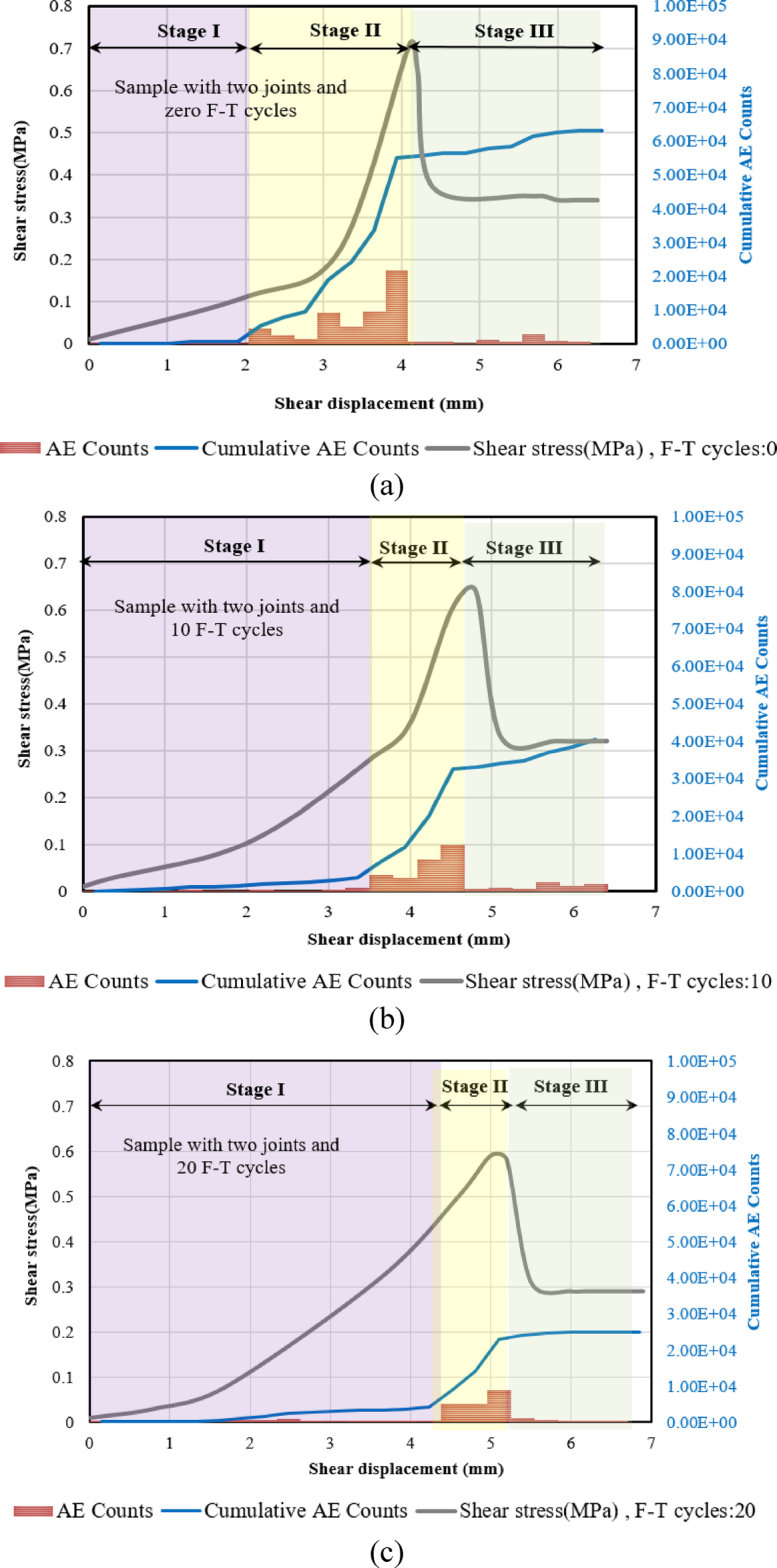
Correlation of shear stress, AE Counts, cumulative AE counts, and shear displacement for samples with two numbers of joints subjected to various F-T cycles, (**a**) Sample not subjected to F-T cycles, (**b**) Sample subjected to 10 F-T cycles, (**c**) Sample subjected to 20 F-T cycles.

**Fig. 19:**
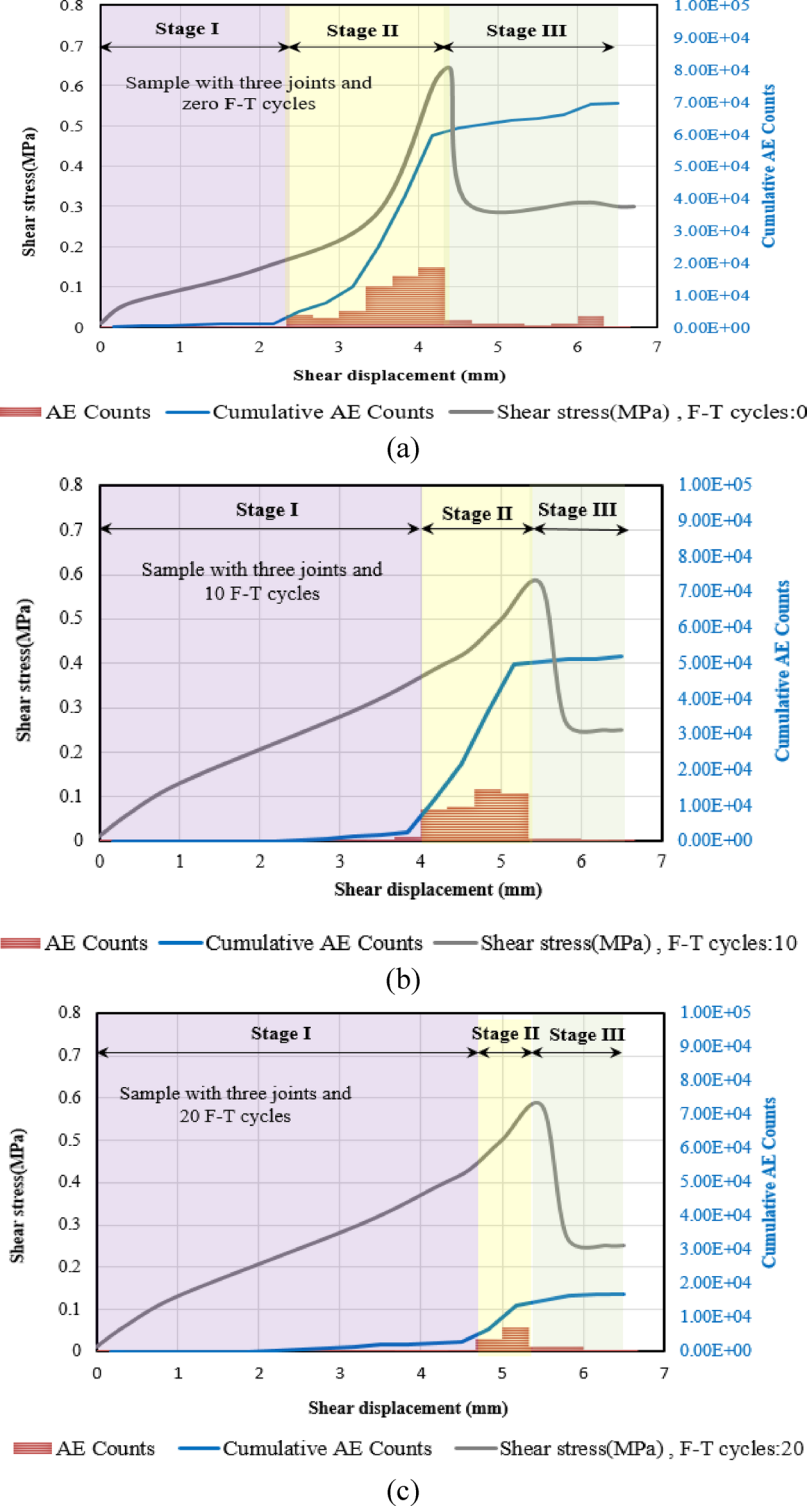
Correlation of shear stress, AE Counts, cumulative AE counts, and shear displacement for samples with three joints subjected to various F-T cycles, (**a**) Sample not subjected to F-T cycles, (**b**) Sample subjected to 10 F-T cycles, (**c**) Sample subjected to 20 F-T cycles.

To explore the crack growth mechanisms of specimens subjected to different F-T cycles, we conducted a detailed analysis of the temporal evolution of AE count and AE energy. Figures 17, 18 and 19 illustrate the progression of acoustic emission counts in relation to shear stress and shear displacement for samples subjected to 0, 10, and 20 F-T cycles, all tested at a 180-degree rock bridge angle under normal stress of 0.3 MPa.

We categorize the direct shear test into three distinct stages, starting with the initiation of loading and concluding with the substantial decrease in shear load. This finding holds for all samples with varying numbers of joints.

##### **Stage I (Quiet Stage**)

Minimal acoustic emission (AE) counts are observed during this stage, attributable to the relatively low shear stress.

##### Stage II (AE development Stage)

As the shear stress continues to increase, AE counts begin to occur sporadically in the beginning. Notably, once AE events are detected, the count rate escalates rapidly, reaching its peak. As the shear stress nears its maximum threshold, there is a significant surge in the AE count rate, with the majority of AE count recorded taking place during this stage. The duration of this stage diminishes with the application of additional cyclic freeze-thaw (F-T) treatments.

##### Stage III (Drop AE Stage)

When the rock bridge fails and the specimen’s peak shear strength transitions to its residual strength, the AE counts abruptly decrease.

**Fig. 20:**
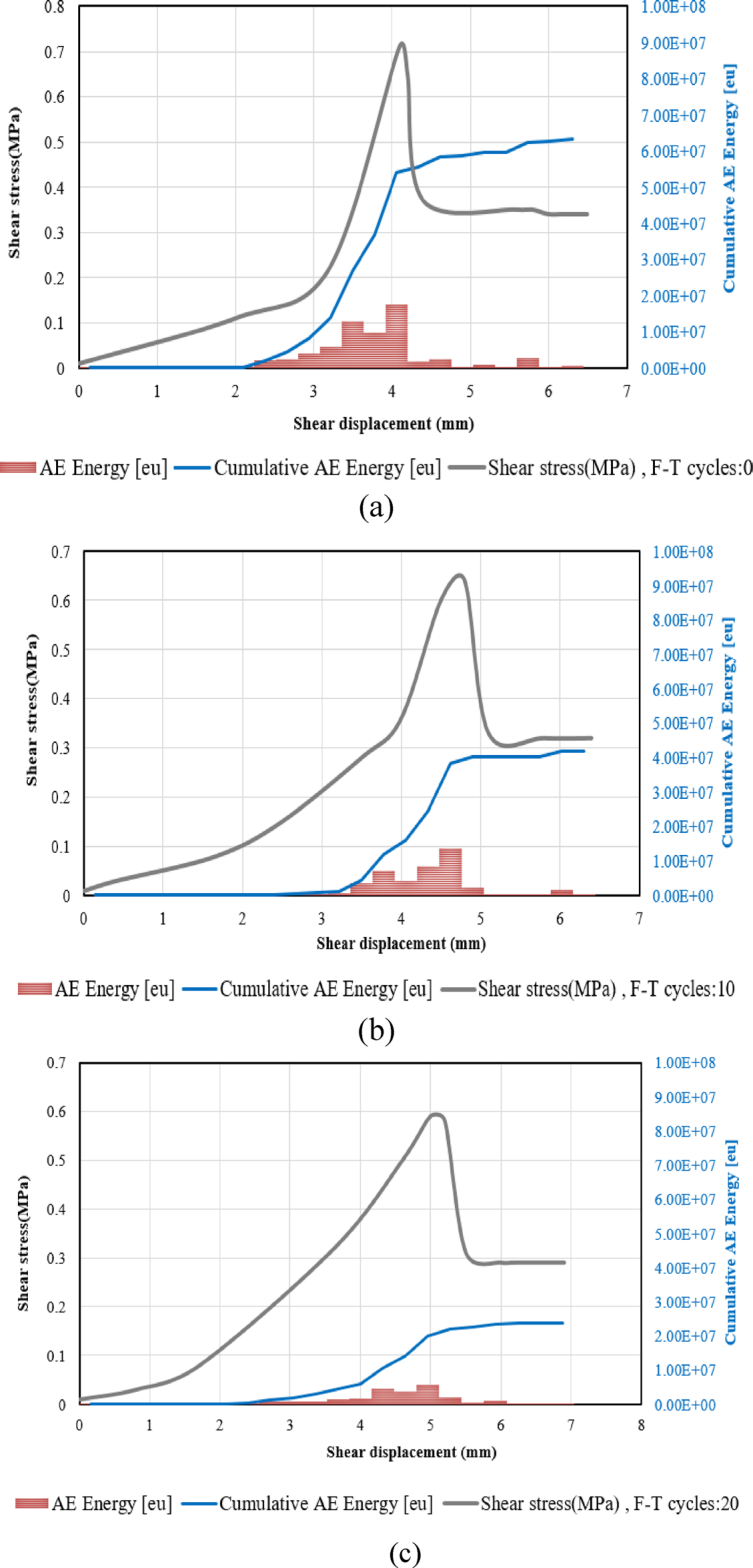
Variations in AE energy for samples with two numbers of joints subjected to various F-T cycles, (**a**) Sample not subjected to F-T cycles, (**b**) Sample subjected to 10 F-T cycles, (**c**) Sample subjected to 20 F-T cycles.

AE energy is another valuable parameter for understanding the behaviors associated with crack initiation, growth, and coalescence. This energy, as recorded by acoustic emission sensors, is typically defined as the area under the envelope of the rectified signal, it is the received amplitude multiplied by the duration of the event. In this investigation, the results were averaged from two different sensors. Figure 20 illustrates the effect of cumulative damage from F-T cycles on the evolution of AE energy in specimens with two joints.

As shown in Fig. 20, the results indicate that the AE energy level decreases as the number of F-T cycles increases. This phenomenon can be attributed to the ongoing deterioration of the particles’ strength and cohesion within the sample as the number of F-T cycles increases. This results in the breakage of some internal particle connections, gradually softening the sample. Consequently, when the sample is exposed to external loads, the accumulated energy is released early, while the ductility of the sample reduces the amount of energy released. As a result, the frequency of AE events decreases during the shearing process. Similar observations have been reported in the research conducted by Wang et al.^27^.

#### Influence of the number of joints

**Fig. 21:**
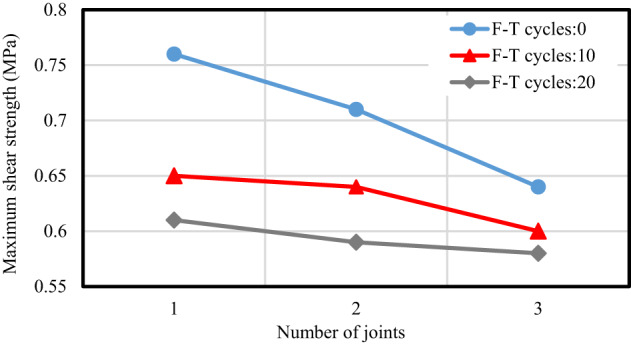
Influence of the number of joints on maximum shear strength across various F-T cycles.

As illustrated in Fig. 9, variations in the number of joints at a consistent percentage (K) exhibit minimal impact on shear strength. The experiments presented in Table 6 and the results illustrated in Fig. 21 indicate that an increase in the number of joints causes a reduction in shear strength. This can be explained by the fact that, in samples with multiple rock bridges, the failure of individual bridges can contribute to the overall reduction in strength. In contrast, a sample with a single rock bridge tends to resist failure in a unified manner. In other words, as the number of rock bridges increases, the likelihood of individual failures also rises. The failure of one or more rock bridges can lead to a decrease in total shear strength. Furthermore, the results indicate that as the number of F-T cycles applied to the samples increases, the variation in shear strength among samples with varying joint numbers diminishes. For instance, in the absence of freeze-thaw cycles, a sample with a single joint displays a shear strength of 0.76 MPa. However, as the number of joints increases to two and three, the shear strength decreases by approximately 7% and 16%, respectively. In contrast, samples subjected to 10 and 20 F-T cycles show a lesser reduction in shear strength versus the number of joints. Specifically, after enduring 20 F-T cycles, the shear strength of the sample with one joint decreases to 0.61 MPa. Meanwhile, samples containing two and three joints experience reductions of 3% and 5% in shear strength under the same freeze-thaw conditions. This phenomenon can be attributed to the deterioration of the samples resulting from the F-T cycles. Therefore, it can be concluded that in samples exhibiting lower strength, the impact of joint numbers at a consistent percentage on shear strength becomes less significant.

**Fig. 22:**
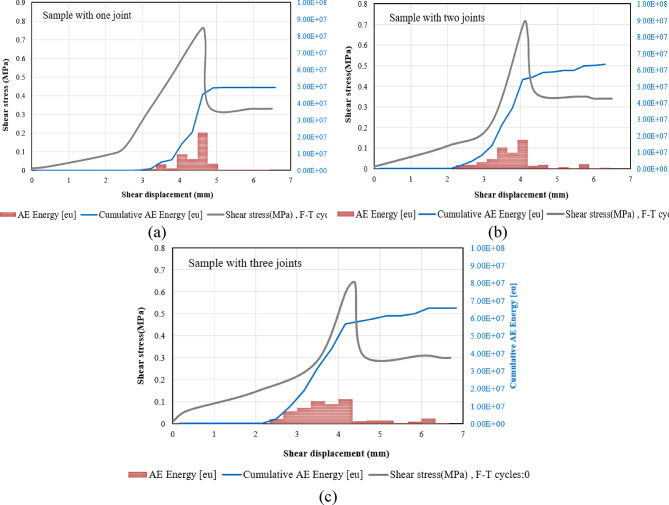
Influence of the number of joints on maximum shear strength across various F-T cycles.

The acoustic emission data indicates that as the number of joints increases, the recorded AE energy levels approach their maximum values, exhibiting only slight variations (see Fig. 22). This phenomenon can be attributed to the fact that shorter rock bridges generate higher stress concentrations around the joints, which in turn increases the likelihood of localized failures. Conversely, longer rock bridges promote more effective load distribution and demonstrate a more uniform failure mode under stress. Consequently, while the initial AE energy may be lower, the moments of failure may yield higher AE energy release events, as the material must fracture through a larger intact portion of the rock.

## Conclusion

In this study, we investigated the impact of various factors, including normal stress (σ_n_), rock bridge angles (β), freeze-thaw cycles (F-T cycles), and the number of joints (N) with equal percentages, on the fracturing and shear behavior of rock-like specimens subjected to direct shear testing. The key findings from this research can be summarized as follows:

1. The results of the P-wave velocity, uniaxial compressive strength, and Brazilian tests suggest that damage to the specimens caused by frost heave is primarily concentrated in the initial stages of the F-T cycles.
2. Based on the experiments conducted using the Taguchi approach and the subsequent analysis of variance (ANOVA), it can be concluded that among the four parameters examined in this study, normal stress has the most significant impact on shear strength, while the number of joints exerts the least influence. The angle of the rock bridge is identified as the second most influential factor affecting shear resistance, followed by F-T cycles in third place.
3. Based on the cracks observed in the rock bridge under shear stress and the data obtained from the acoustic emission method, it can be concluded that a rock bridge angle of 90 degrees results in tensile failure. As the rock bridge angle increases to 180 degrees, the failure mode transitions from tensile to shear. For angles between these two values, a combination of tensile and shear failure occurs.
4. Based on the acoustic emission data, the direct shear test for specimens subjected to F-T cycles can be categorized into three distinct stages: a quiet stage, an AE development stage, and a drop AE stage. As the number of F-T cycles increases, the duration of the AE development stage and the AE energy level diminish. Additionally, as rock bridge length decreases, the recorded AE energy levels during the test approach their maximum values. Conversely, longer rock bridges are associated with lower initial AE energy release, while the moments of failure tend to yield higher AE energy release events.

The results obtained from this research will be applicable at larger scales and in real-world projects, enabling engineers to mitigate shear failure risks, predict failure modes, and enhance stability assessments of rock structures. These findings also support sustainable engineering designs that can enhance local community safety and environmental sustainability in freeze-thaw-prone regions. These advancements improve the longevity and safety of geotechnical structures, particularly in freeze-thaw-prone regions. Furthermore, this study provides a foundation for investigating the effects of rock heterogeneity, extreme pore pressure fluctuations and dynamic loading rates on rock behavior with non-persistent joints, which are essential for addressing saturation conditions and seismic challenges in future research.

## Data Availability

The data that support the findings of this study are available from the corresponding author upon reasonable request.
